# Inflammatory Cytokines Shape an Altered Immune Response During Myeloid Malignancies

**DOI:** 10.3389/fimmu.2021.772408

**Published:** 2021-11-03

**Authors:** Virginia Camacho, Valeriya Kuznetsova, Robert S. Welner

**Affiliations:** Department of Medicine, Division of Hematology/Oncology, O’Neal Comprehensive Cancer Center at the University of Alabama at Birmingham, Birmingham, AL, United States

**Keywords:** cytokines, leukemia, immune cells, microenvironment, inflammation

## Abstract

The immune microenvironment is a critical driver and regulator of leukemic progression and hematological disease. Recent investigations have demonstrated that multiple immune components play a central role in regulating hematopoiesis, and dysfunction at the immune cell level significantly contributes to neoplastic disease. Immune cells are acutely sensitive to remodeling by leukemic inflammatory cytokine exposure. Importantly, immune cells are the principal cytokine producers in the hematopoietic system, representing an untapped frontier for clinical interventions. Due to a proinflammatory cytokine environment, dysregulation of immune cell states is a hallmark of hematological disease and neoplasia. Malignant immune adaptations have profound effects on leukemic blast proliferation, disease propagation, and drug-resistance. Conversely, targeting the immune landscape to restore hematopoietic function and limit leukemic expansion may have significant therapeutic value. Despite the fundamental role of the immune microenvironment during the initiation, progression, and treatment response of hematological disease, a detailed examination of how leukemic cytokines alter immune cells to permit, promote, or inhibit leukemia growth is lacking. Here we outline an immune-based model of leukemic transformation and highlight how the profound effect of immune alterations on the trajectory of malignancy. The focus of this review is to summarize current knowledge about the impacts of pro- and anti-inflammatory cytokines on immune cells subsets, their modes of action, and immunotherapeutic approaches with the potential to improve clinical outcomes for patients suffering from hematological myeloid malignancies.

## Overview of Myeloid Malignancies

Myelodysplastic and myeloproliferative neoplasms (MDS/MPNs) are highly heterogeneous clonal blood disorders that vary in cellular composition, genetic pathology, and disease course. Despite their structural and molecular differences, myeloid malignancies share several commonalities in biology. Their underlying cellular origin is typically transformed hematopoietic stem or progenitor cells (HSPCs) which outcompete the healthy counterparts. The clonal nature of these disorders arises from an uncontrolled expansion of differentiation‐blocked cells that cannot progress to mature lineages (1–3). This skewing creates defects in hematopoietic output, eventually leading to clonal expansion and leukemic progression. Moreover, normal hematopoietic cells are forced into competition with mutant leukemic cells as the two populations occupy the same microenvironment. Other hallmark phenotypes include the uncontrolled production of myeloid cells, excessive inflammatory signaling, and immune exhaustion (4, 5). These findings invite questions into how immune changes synergize with niche alterations to promote disease initiation at the expense of healthy hematopoiesis. Thus, strategies to rejuvenate the immune response and curb associated inflammatory signaling represent an exciting path for treating these disorders.

An early distinction of different MPN entities is between Philadelphia chromosome-negative (-) and Philadelphia chromosome-positive (+) malignancies. Essential thrombocythemia (ET), polycythemia vera (PV), and primary myelofibrosis (PMF) represent the three classical Philadelphia chromosome-negative (-). They are characterized by exaggerated platelet production, increased thrombosis and clotting, and tissue fibrosis respectively. In these MPNs the cytogenetics are characterized by mutations in either Janus kinase *(JAK) 2* gene, myeloproliferative leukemia virus proto-oncogene *(MPL)*, Calreticulin *(CALR)*, and colony-stimulating factor 3 receptor *CSF3R.* Although driver mutations in JAK2 are the most common (approximately 70% of MPNs) the other mutations also hyperactivate the JAK-STAT pathway or directly promote abnormal cell proliferation and differentiation (6).

In Essential thrombocythemia (ET), persistent elevated platelet counts and megakaryocytic proliferation in the marrow are hallmark features with nearly 55% of patients harboring the *JAK2 V617F* mutation. Thrombotic events and transformation to more aggressive myeloid disorders AML or myelofibrosis are risk factors. Virtually all patients with Polycythemia vera (PV), harbor a JAK2 mutation, and 14–20% of individuals with carry a karyotype abnormality. Clinical features include increased red blood cell production, low EPO and increased likelihood of thrombotic complications. Primary myelofibrosis (PMF) is characterized by bone marrow fibrodysplasia, anemia, splenomegaly. While the *JAK2*, *CALR*, and *MPL* are the most represented genetic lesions in PMFs, recent advances have revealed that mutations in epigenetic regulators, RNA splicing machinery, and DNA methylation genes are more commonly seen in PMF than ET and PV with this MPN also having a lower median survival rate. Interestingly, mutations in Additional sex combs like 1 (ASXL1) are over-represented in PMF patients and may contribute to risk stratification for this cohort and the dysplastic phenotypes (6–8).

Chronic myelogenous leukemia (CML) is a hematopoietic stem cell disorder that results from the translocation of the tyrosine-protein kinase (*ABL1*) on chromosome 9 to the breakpoint cluster region (*BCR*) gene on chromosome 22. This creates an oncogenic fusion gene (BCR-ABL1) with persistently enhanced tyrosine kinase activity (9). In recent years, tyrosine kinase inhibitors (TKIs) have dramatically improved quality of life and survival outcomes with many patients achieving deep molecular response (DMR) and molecular remission even after TKI discontinuation (10, 11). Similarly, several immune cell states and cytokine signatures have emerged as prognostic indicators of disease outcome and seem to play a role in achieving DMR in CML patients on TKI therapy (12).

### Myelodysplastic Syndromes

Myelodysplastic syndromes (MDS) represent a heterogeneous group of hematological malignancies. While numerous phenotypes are associated with MDS pathology, epigenetic alterations, cytopenia, cellular dysplasia, and acute risk of leukemic transformation are hallmark traits of the disease. Recently, various molecular abnormalities including somatic mutations and cytogenetic lesions have been closely associated with MDS pathology. Candidate-gene screens and Genome Wide Association Studies have shown that enzymes regulating the modification and methylation of DNA are frequent mutated across MDS patients (13, 14). The most common alterations involve mutations in *DNMT3A*, *TET2* and *ASXL1*. Collectively these epigenetic modifiers catalyze the modification of methyl groups to cytosine residues of CpG dinucleotides, modify chromatin marks, and shape the methylation patterns that promote differentiation into different lineages. Although they differ in biological function mutations in these genes tends to produce convergent phenotypes including progressive multi-lineage cytopenias, cellular dysplasia, increased numbers of hematopoietic stem and progenitor cells (HSPCs), and a pleiotropy of differentiation defects. Additional recurrent molecular abnormalities associated to CHIP include mutations in genes encoding for RNA splicing factors, (*SF3B1*, *SRSF2*, *U2AF1*, and *ZRSR2* genes) (15). Of note, clinical studies have demonstrated that the two classifications of MDS vary significantly in their niche profiles. Furthermore, massive parallel RNA sequencing of purified MSCs from low-risk MDS patients reveals that these cells have a genetic signature of cellular stress and inflammation which is functionally and molecularly distinct from their normal counterparts (16).

Recent investigations have shown the initiation of MDS and potential transformation to acute myeloid leukemia (AML) is strongly linked to chronic inflammatory conditions (17). Indeed, epidemiological investigations have demonstrated a clinical association between antecedent auto-inflammatory and autoimmune diseases and MDS development (1, 18). However, extensive modeling on somatic evolution is needed to determine if immune alterations predate leukemic initiation or if pre-existing intrinsic immune dysfunction is an initiating driver in the development of dysplastic disease.

Overall, MDS patients present with various alterations in cellular and cytokine-mediated immunity, and there is increasing evidence that immune cells contribute to MDS pathology. Regarding adaptive immunity, oligoclonal T cell expansion and increases in T lymphocytosis have been documented in about 50% of the MDS patients (19). Defective NK-cell-mediated cytotoxicity, distorted antibody and cytokine production, neutrophil dysfunction, and DC alterations have also been described. Bone marrow failure represents a significant risk in MDS, and while the pathological features are complex, T cells are thought to drive tissue pathologies such as excessive proliferation and apoptosis of marrow cells. Despite these findings, current risk assessment and management of MDS is primarily focused on the cytogenetic constituents of the disease, overlooking specific cellular actors and immunological components.

### Acute Myeloid Leukemia

Acute myeloid leukemia (AML) is characterized by genetic lesions, chromatin re-arrangements, and epigenetic modifications that result in arrested differentiation, and increased leukemic blast proliferation and survival. Although AML prognosis is highly variable, the high rates of relapse and mortality pose a significant clinical burden. The WHO classification subdivides AML into six categories based on genetic, morphologic, clinical and immune phenotypes. These include (1) AML with recurrent genetic abnormalities, (2) AML with myelodysplasia-related changes, (3) Therapy-related AML, (4) AML not otherwise specified, (5) AML with myeloid sarcoma, and (6) AML proliferations related to Down syndrome (DS) (20). The overwhelming majority of AML cases are associated with chromosomal translocations that form oncogenic fusion proteins, but despite AML having a lower mutational burden than other adult cancers, the disease is marked by extreme molecular heterogeneity. Therefore, it is likely that the flavor of immunological events contributing to AML development is subtype specific and informed by the mutational background of individual patients. As with the other MPNs, excessive inflammatory signaling is a dominant feature, and individuals with chronic immune-activating and autoimmune diseases are at increased risk for AML development (21). Common immune phenotypes include, effector T-cell exhaustion, Treg expansion, reduced NK cell activity, and dysregulated expansion of various myeloid subsets. Disruption of immune checkpoint mechanisms including the downregulation of major histocompatibility complex (MHC) class I and class II molecules, and upregulation of immune checkpoint regulators (PD-L1 CTLA-4 and LAG-3) are also hallmark traits (22). The propensity for other MPNs to develop into AML invites more investigations into which pre-existing immunological features accelerate the onset of AML progression. Likewise, an analysis into how cooperating mutations in AML distort myeloid cell identity is an incompletely characterized space. Given the reliance of AML blast on the local cytokine environment unraveling the molecular basis for these hyperinflammatory immune phenotypes, which manifest as increased secretion of inflammatory mediators, cytokines and growth-factors, should be further investigated.

### Clonal Hematopoiesis of Indeterminate Potential

Clonal hematopoiesis of indeterminate potential (CHIP) presents as somatic mosaicism in the blood, arising from clonal expansion of hematopoietic cells harboring one or more genetic variations (23). Mutations in epigenetic regulators, specifically enzymes regulating the modification and methylation of DNA such as *DNMT3A*, *ASXL1*, *TET2*, represent the majority of CHIP mutations (24). These alterations can be founder events that increase individual risk of more severe diseases such as MDS and AML (25). There has been significant and elegant work characterizing the effects of dysregulated inflammatory cytokine signaling on competitive advantage and clonal evolution of HSCs harboring mutations (26, 27). However, it is now appreciated that CHIP also increases all-cause mortality. The presence of somatic mutations on immune progeny influences the pathology of other conditions, including atherosclerosis, infections, cardiovascular disease, and chronic obstructive pulmonary disease (COPD) (28–31). Elevated levels of IL-6/IL-1β are hallmark traits across these non-cancerous diseases. To date, the best characterized cellular source of these cytokines are myeloid cells harboring CHIP mutations. CHIP-associated mutations in the myeloid compartment prime these cells for hyper-inflammatory responses, impair cellular function, and increase the release of inflammatory mediators (32, 33). Nevertheless, the effects of these mutations on homeostatic functions such as tissue repair and niche maintenance are still lacking. Even less is known about how CHIP- mutations influence adaptive immunity. Unlike the myeloid compartment, somatic mutations are infrequently present in the adaptive immune compartment, making lymphocytes bystanders of inflammatory cytokine remodeling. The absence of somatic mutations in the adaptive arm is an age-related consequence. Active thymopoiesis (dominating in the early decades of life) and clonal hematopoiesis (accelerating with increasing age) do not share significant temporal overlap. Nonetheless, myeloid cells are necessary for adaptive immune licensing, underscoring the importance of altered myeloid interactions on a range of immunological functions, including priming, antigen presentation, T cell exhaustion, immunological memory, and tissue maintenance.

## Cytokine Signaling in Hematological Disease

**Figure 1:**
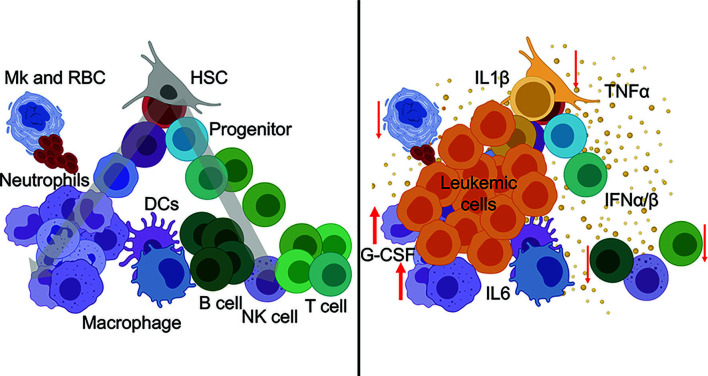
Hematopoietic perturbation from inflammatory cytokines during leukemia. (Left) Normal hematopoiesis is a continuous process from stem cells (top) through mature immune lineages (bottom). (Right) Leukemia-associated inflammatory cytokines mediate the differentiation, abundance, and cellular functions of innate and adaptive immune populations during disease.

The cytokine environment is a critical determinant of immune function, both in steady-state and in disease. Cytokine exchanges between innate and adaptive immune cells and supporting stromal counterparts are essential for maintaining organismal homeostasis and tissue-specific regional immunity. Cytokines are potent soluble proteins that mediate immune cell fate, immune cell function, and immune-mediated pathologies. Cytokines exert their effects in highly localized environments as well as systemically. In myeloid malignancies, a link between heightened inflammatory signaling and disease progression has been established. Notably, chronic immune-mediated inflammation is now considered an early trigger and driver of clonal evolution in pre-leukemic states. Cytokine profiling of patients with CHIP, MDS, and AML points reveals expression of many inflammatory factors. Among the various elevated cytokines and chemokines, IL-1β, IL-6, IFN-γ, and TNF-α have established roles as immunomodulators of leukemic progression (2, 5). *Refer to* **Figure 1** *for perturbation of immune populations during hematological malignancies.*

### Proinflammatory Mediator IL-1β

IL-1 is a proinflammatory cytokine produced by myeloid cells and monocytes in response to infection and cellular stress and various investigations have uncovered the role of IL-1β in HSC and myeloid cell homeostasis (34, 35). Many studies have stressed the importance of IL-1 in hematological malignancies, and elevated levels of IL1β levels are seen in CHIP, MPNs, MDS, and AML (36). In CML, increased IL-1β predicts a poor prognosis. Consistent with this, in CML murine models, IL-1 receptors are elevated on the surface of LSCs, underscoring a potential dependency on these inflammatory signals (37, 38). As with many of these cytokines, the role of IL1β in AML is varied, likely due to the diversity of subtypes within the disease. Several studies have indicated a role for IL-1β in the expansion of AML *via* p38 MAPK phosphorylation. Furthermore, increased levels of IL-1β and IL-1 receptors have been reported in AML patients, and AML blasts have been shown to secrete IL-1β themselves (39, 40). More intriguing are studies that target IL-1 signaling pathways in MPNs and models of clonal hematopoiesis. This is particularly prevalent in models where *TET2* is mutated and mutant macrophages have increased IL-1β secretion (31, 41–43). With recent studies showing increases in IL-1β expression in CHIP and MDS, clinical trials targeting this cytokine pathway are perhaps upcoming.

### Proinflammatory Mediator IL-6

IL-6 is an inflammatory cytokine secreted by monocytes and macrophages, and sometimes T cells that uniquely drives the influx of neutrophils and other myeloid cells to the site of secretion (44). IL-6 plays an important role in CML, exerting its modulating effects on leukemic HSC as well as in bystander cells in a manner that promotes disease progression (45, 46). Increased IL-6 levels drive LSC expansion and associated pathologies. Investigations that have uniquely studied the role of this cytokine highlight its importance in pathogenesis (2, 47). Interestingly, the aged microenvironment secretes higher levels of IL-6 that promote HSC dysfunction and support of leukemic cells at the expense of health counterparts (48). Since aging is a component of many of these malignancies, these observations neatly intersect with the increased levels of IL-6 that accompany the aging process. In individuals with CHIP, genetically reduced IL-6 signaling confers protection against CHIP-associated pathologies (49). Efforts are underway to target this cytokine in MDS and MPNs, given the predictive nature of this cytokine in disease outcomes. These discoveries indicate that IL-6 overexpression fuels the acquisition of more aggressive leukemic phenotypes *via* the immune microenvironment and that targeting this pathway could delay progression and reduce activation of oncogenic signaling.

### Effector Cytokine TNF-α

TNF-α can be produced by multiple immune subsets (T cells, NK cells, neutrophils), AML blasts, but macrophages and monocytes are the prominent source of this effector cytokine during inflammation (50, 51). TNF-α signaling contributes to a diverse range of biological events but canonically stimulates dividing cells’ proliferation and induces necrosis or apoptosis. Thus, it has a dominant role in regulating cell growth and cell death.

In MDS, where pancytopenia and hypercellularity are distinguishing features, elevated levels of TNF-α in both the serum bone marrow have been reported (52, 53). Additionally, polymorphisms in the human *TNF* gene that increase TNF-α levels are overrepresented in newly diagnosed MDS patients, and neutralization of this cytokine has been shown to restore hematopoiesis (54, 55). A possible explanation is that increased TNF-α stimulation exacerbates T cell mediated cellular apoptosis and peripheral cytopenia thus driving MDS pathogenesis.

TNF-α is also increased in AML, and its effects on downstream NF-kB signaling help maintain LSCs (56–58). Interestingly, when AP1 transcription factors are co-activated, these signals synergize to promote proliferation and survival of leukemic cells (59). In these cases, it is likely that inhibition of TNF-α in combination with modulation of other cytokines is needed for the eradication of the leukemia-initiating cells. Several ongoing clinical trials are underway to target TNF-α signaling alone or in combination with other activating and inhibiting pathways.

### Immunomodulating Interferons

Interferons (IFNs) are important for HSC biology, with distinct roles for type I and type II IFNs (5, 60). IFNα and IFNβ are the best-defined type I family members. They are produced ubiquitously in response to the stimulation of cell surface and intracellular pattern recognition receptors, such as TLRs. Historically, interferons were named for their ability to ‘interfere’ with viral replication as part of the host defense response. IFN-α/β signals influence innate and adaptive immunity at many levels, but they are crucial for inducing antiviral states and host protection against viral infection (61). IFN-α/β signals also potently inhibit cellular responsiveness to IFN-γ, highlighting suppressive and regulatory aspects of type I IFNs (62). The type II family member, IFN-γ has potent activating effects on the innate response, particularly on macrophage polarization. Despite the shared nomenclature and synergistic effects, type I and II IFNs signal through different receptors. Moreover, IFN-γ is predominantly produced by activated lymphocytes (T cells and NK cells) and is more closely associated with anti-tumor mechanisms as well as growth and differentiation (63).

Interferon therapy has a history of clinical application across MPNs and administration of IFN-α has been shown to arrest differentiation and to exert anti-leukemic effects. CML cells treated with IFN-α show increased apoptosis through the p38 MAPK pathways (64, 65). These proliferative effects provide a therapeutic window for sensitizing LSCs to chemotherapy and combinatorial approaches are being pursued in several clinical trials. With regards to IFN-γ, reports have shown that IFN-γ favors LSCs and contributes to TKI resistance (66). Meanwhile, in PV, IFN-γ leads to increased proliferation of HSC carrying these mutations, and this loss of dormancy results in selective targeting of leukemic HSC compared to healthy bystanders (67, 68).

With respect to MDS, *DNMT3A* deficient cells secrete more IFN α/β, and recent evidence suggests that IFN signaling promotes selective expansion of clones carrying *DNMT3A* mutations. It is likely that increases in IFN-γ come from expanded CD8^+^ T cells in MDS patients and contribute to disease progression through STAT1 activation (4, 69). To further complicate the role of IFN-γ, this cytokine was shown to promote MDS and AML differentiation into effector innate immune lineages (70–72). On a similar note, ET or PV patient derived erythroid colonies have increased IFN-γ and STAT1 expression (73). Given the pleiotropic nature of IFNs in MDS and AML, the use in the clinic is limited and requires further categorization for specific targeting. Nonetheless, functional IFN-γ-mediated responses are still positively associated with patient survival and prognosis, highlighting the need to understand the regulatory mechanisms by which IFN signaling modulates the immune response in leukemic settings.

### Anti-Inflammatory Mediator IL-10

IL-10 has diverse roles in the context of inflammation, regeneration, and cellular communication. The ubiquity of IL-10 expression and the conservation of its regulatory functions across vertebrate species highlight the evolutionary importance and multifunctional nature of this cytokine (56). The ability to integrate IL-10 signals is found in hematopoietic and non-hematopoietic cells underscoring its biological relevance (74). Nearly all immune cells can produce IL-10 or respond to IL-10 signals through corresponding receptors (IL-10R1) and IL-10R2 (75, 76). IL-10 is a ‘Type II cytokine’ branded by the original studies on IL-10 in shaping the humoral immune response and its association as an inhibitory T-helper 2 (Th-2) cytokine (77). At the basic level, IL-10 signals through the Janus kinase (JAK) and signal transducer and activator of transcription (STAT) signaling pathways. Downstream integration of this signal is both context-dependent and cell type-specific involving layers of genomic, epigenetic, and transcriptional regulation (78).

While the roles of IL-10 are vast, it is principally recognized for its suppressive functions including the inhibition of cellular activation and downregulation of inflammatory responses. The expression profile of IL-10 varies significantly in myeloid malignancies, and its role in disease progression remains controversial. Disease-promoting effects *via* the reduction of anti-tumor and anti-leukemic have been reported as have beneficial anti-inflammatory properties. The suppressive properties of IL-10 have reinforced the assumption that IL-10 limits the ability of the immune system to eradicate malignant clones. In AML, for example, targeting the IL10R on leukemic blasts *via* chimeric antigen receptor (CAR) T cells had beneficial effects, increasing anti-tumor cytotoxicity (79). Nonetheless, murine and human studies have revealed that defects in IL-10 signaling increase the incidence of early cancer development (80). Mechanistically, IL-10–activated STAT3 also activates the suppressor of cytokine signaling 3 (SOCS3), which subsequently inhibits proinflammatory effects of IL-6 and IL-12/IL-23 receptors. Therefore, it is likely that the efficacy for IL-10 might be best targeted in the early stages of diagnosis, where this cytokine could interrupt inflammatory cascades and alleviate leukemia-promoting inflammation.

## Overview of Immune System in Steady-State

The immune microenvironment is a critical determinant of leukemic transformation and a driver of progression to more aggressive leukemias. In contrast to solid tumors, with spatially and anatomically constricted metastasis, blood cancers pose a unique immunological challenge. First, the presence of oncogenic mutations in hematopoietic stem and progenitors (HSPCs) leads to the inheritance of functionally disruptive mutations in mature immune cell progeny. Additionally, leukemic cells circulate freely throughout the body, interacting with multiple microenvironments and reshaping multiple tissue niches simultaneously. Mechanistic investigations into the influence of leukemic cytokines on myeloid and lymphoid immune function and the reciprocal effects of altered immune-mediated cytokine signaling on leukemic evolution have the potential to inform the implementation of future immunotherapies in leukemic disease.

The immune system is classically divided into two compartments: an innate and adaptive arm, bifurcated according to functional diversities (81). The innate compartment functions as the initial line of defense in response to foreign signals from viruses, microbial invasion, or tissue injury. This sophisticated sentinel system differentiates itself from the adaptive arm principally by its rapid response time and broad sensitivity that evolved to counter the accelerated doubling time of foreign pathogens. The cellular components of the innate immune system are ubiquitous throughout tissues but have a significant presence at barrier sites and mucosal surfaces. Specialized myeloid and phagocytic cells (monocytes, macrophages, neutrophils), antigen-presenting cells, (dendritic cells), cytotoxic lytic cells (NK cells), subsets of T cells with alternative antigen receptor diversity (invariant natural killer T cells, *γ*δ T cells), and non-hematopoietic cells (epithelial cells), can recognize and process danger signals in response to stress, damage or infection and initiate rapid immune responses. Notably, patients with myeloid malignancies have increased susceptibility to infection, and infection-related complications are a significant risk factor and cause of death among these groups. Patients also present with suboptimal immune responses following vaccination (82). In line with this, MPN and MDS patients often suffer from neutrophil and monocyte dysfunction, likely contributing to decreased viral immunity, increased risk of tissue damage, and increased organ dysfunction under viral stress.

In contrast to innate immunity, the adaptive system is characterized by its robust specificity. While the innate system relies on germ-line encoded recognition molecules to sense structural patterns on foreign molecules, the generation of antigen-specific receptors (T- and B-lymphocytes) gives the adaptive arm significant power of precision. This specificity allows the adaptive arm to mount rapid and effective responses after repeated exposures with the same antigen and to confer protection at an extended timescale (i.e., the lifetime of an organism). T cell lymphocytes (T-cells) are critical actors within the adaptive arm contributing to immunological memory, and barrier tissue-immunity (83). T cells are antigen-specific cells. Upon activation and exposure to their cognate antigen, they undergo maturation and clonal expansion, or become long-lived memory cells poised for future recall responses (84).

## Innate Immunity in Myeloid Malignancies

Myeloid cells provide immunomodulatory cytokine signals and necessary cell–cell contacts to tissue niches, adaptive immune cells, and leukemic blasts thereby significantly contributing to immune escape and leukemic niche remodeling. Myeloid cells harboring different oncogenic mutations are also poised for increased proinflammatory cytokine secretion, increased production of ROS, and aberrant immune activation representing an additive force in leukemic disease progression and a confound for immunotherapy.

### Dendritic Cells

Dendritic cells (DCs) are professional antigen-presenting cells that prime adaptive immune responses through co-stimulatory cell-cell contacts and cytokine exchanges. DCs are highly heterogeneous and are broken down into developmentally and functionally distinct populations (85). Different DC subsets can induce strong adaptive immune responses to foreign antigens or conversely to promote induction of tolerance to self-antigens (86). Flt3 Ligand (Flt3L) is critical for DC commitment in hematopoiesis, as evidenced by dramatic decreases in DC populations in the absence of active Flt3 signaling. Notably, Flt3 activating mutations are frequently found in AML patients and are associated with poor prognosis. Despite constitutive activation, evidence suggests that FL may continue to play a signaling role, but the effects of constitutive activation on DC development and function in AML are still unknown (87).

In combination with antigen presentation, DC co-stimulatory signals, and DC-derived cytokines, (type I interferons, and IL-12) are critical determinants of T cell effector function. DCs can exacerbate and sustain chronic inflammation by activating antigen-specific T cells, as has been described in various autoimmune conditions. It is also likely that alterations to DC function may induce T cell anergy or exhaustion and suppress desired anti-tumor responses when paired with a prolonged inflammatory environment (88, 89).

Myeloid “inflammatory” DCs are potent producers of various inflammatory mediators, including TNF-α, reactive oxygen species (ROS), such as inducible nitric oxide synthase (iNOS), and IFNα/β, which have all been implicated in leukemic disease. Monocytes of patients with J*AK2V617F* mutations display increased and sustained production of TNF-α following stimulation with TLR agonists, LPS (TLR4), and R848 (TLR7/8) (90). Of note, MPN monocytes also had a blunted response to IL-10 despite producing similar levels of IL-10 upon stimulation, indicating defects in critical negative feedback regulation. Increased endogenous ROS and reactive nitrogen species (RNS), such as iNOS, as well as high expression of plasma MDA (a by-product of lipid peroxidation) and PC levels (product of oxidized proteins), are also common features.

### Macrophages

An essential macrophage function is to engulf foreign agents and these cells represent the principal phagocyte of the immune system. Macrophages are recruited to sites of injury or inflammation and are activated by contact with pathogens or danger signals (activation of pattern recognition receptors or inflammasome). They support organismal homeostasis by eliminating apoptotic cells, recycling nutrients, and digesting waste products from tissues. Macrophages are highly plastic, and their heterogeneity reflects functional specialization in response to different cytokine and tissue environments. While many intermediate and diverse phenotypes have been described, the two historical subsets are defined as classically activated M1 (proinflammatory type 1), and alternatively activated M2 (anti-inflammatory type 2), representing opposite ends of the functional spectrum (91). The M1 and M2 states are defined by their immunomodulatory properties, transcriptional profile, surface makers, and morphology. When exposed to inflammatory stimuli, macrophages secrete several cytokines, including inflammatory mediators such as TNF-α, IL-1β, IL-6, IL-8, and IL-12 (92). Many of these cytokines, specifically IL-1β and IL-18, are released in active form by activating the inflammasome complexes. This promotes leukocytes migration, and kickstarts the production and activation of neutrophils. At the other extreme of the continuum, anti-inflammatory reparative macrophages promote healing responses and alleviate tissue pathology. This is partially executed by activating IL-10 and TGF-β1 producing Treg cells, which temper damaging inflammatory responses (93).

Various investigations have documented abnormal macrophage polarization in the marrow of MPN and MDS patients (32). An analysis of bone marrow macrophages in different MPNs (CML, PMF, PV, and ET) revealed that PMF patients have increased macrophage frequencies followed by PV and ET (94–96). In CML, a high frequency of CD68^+^, CD163^+^, and CD206^+^ M2-like macrophages was associated with LSC survival and progression to accelerated phase blast crisis (97). This suggests that macrophages have a central role in pathogenesis.

### Neutrophils

Neutrophils are the first line of defense against infectious pathogens and microbes. The lifecycle and differentiation of neutrophils is regulated by balancing their short half-life in the blood with mobilization out of the bone marrow. Chemotherapy-induced neutropenia and neutrophil dysfunction are common in MDS and MPNs, heightening predisposition to infectious disease. Granulocyte colony-stimulating factor (G-CSF) is critical for release, survival, and maturation of neutrophils, and treatment *in vivo* with G-CSF is an effective strategy to increase absolute neutrophil and restore neutrophil function (98).

Currently, we lack causal information on how G-CSF administration modulates leukemic disease progression, and it remains to be determined if the synergism of G-CSF with other cytokines directly influences the acquisition of additional cytogenetic lesions. In models of humanized AML, it has been demonstrated that IL-3/GM-CSF stimulation is required for the transformation of human hematopoietic cells to AML blasts, and individual patients have shown a reversal from a monoclonal to a polyclonal pattern of MDS with GM-CSF therapy (99). Relatedly, severe congenital neutropenia (SCN), a disease marked by increased apoptosis of neutrophils and neutrophil precursors, and patients are treated with recombinant G-CSF (rG-CSF) are at increased risk of leukemogenesis (100).

### Natural Killer Cells

Natural killer (NK) cells are large granular lymphocytes that induce the apoptosis in target cells. NK cells lack rearranged antigen-recognition receptors, and their activity is modulated by an interplay between signals from inhibitory (KIRs, NKG2A, and LIR1) and activating (NKD2D, NCRs, 2B4, DNAM-1) surface receptors. Many inhibitory NK receptors bind HLA-I molecules expressed on healthy cells, thus restraining NK cytotoxicity against the host. In viral infection or malignant transformation, affected cells decrease HLA-I expression and acquire surface stress-associated molecules, common ligands for NK cell activating receptors, triggering NK cell-mediated cytotoxicity.

NK cells are profoundly altered in MDS, AML, and CML but data are inconsistent. Some studies report severely decreased counts in AML and untreated CML patients (101, 102). In line with this, NK cells from peripheral blood of AML patients have defects in synapse formation, expression of activating receptors, and production of IFN-γ, perforin, and granzyme B (103, 104). Conversely, the upregulation of co-inhibitory molecules (PD-1, TIGIT, CD94, TIM-3) deployed by leukemic cells to evade immunity has been reported (105, 106). Similarly, NK cells display low expression of NCR, NKG2C, NKG2D in CML, which is rejuvenated post-TKI (101, 107, 108). Expression of inhibitory molecules (PD-1, TIGIT) appear to be elevated in *de novo* CML, and these levels change in individuals achieving a molecular response (109, 110). A significant decrease in NKG2D and DNAM-1 expression was observed in MDS, and was associated with the impaired killing of MDS blasts (111). Along these lines, increased expression of TIGIT was associated with poor cytokine production and degranulation in blood and marrow NK cells from MDS patients (112).

The ability of NK cells to elicit anti-leukemic responses has been observed both *ex vivo* and in AML patients following allogenic bone marrow transplantation (113, 114). Nonetheless, defective in cytotoxicity and degranulation in common in leukemic disease. This is important, given that cytotoxic granule-mediated cell lysis is the primary mechanism of NK cell anti-leukemic activity. Impaired cytotoxicity aligns with the defects in NK cell maturation reported in CML, MDS, and AML (115–117). The two main NK subsets in humans are pre−mature CD56^br^CD16^low^CD57^-^ with a great potential to produce cytokines, and mature highly cytotoxic CD56^dim^CD16^hi^CD57^-/+^ cells, which possess powerful killing machinery. The mature population appears to be most affected in myeloid leukemias suggesting a loss of the most potent leukemia eradicators. In CML, functional NK cells are likewise associated with successful relapse-free survival upon TKI withdrawal (102, 115, 118). Consistent with human data, skewing of NK cells toward immature phenotype has been observed in MLL/AF9 and MLLPTD/Flt3ITD AML mouse models (119, 120).

Leukemic cytokine imbalances likely disrupt NK cell function. Some have reported an inverse correlation between IL-32 and TNF-α levels and NK cell numbers in bone marrow samples of MDS patients, as well as an increase of these cytokines during the progression to secondary AML (121, 122). This suggests that inflammatory cues impair NK cell differentiation compromising immune surveillance. Several studies have shown an inhibitory effect of IL-6 on NK cell killing capacity in the context of solid tumors, obesity, heart failure, arthritis, and *T. gondii* infection (123, 124). Given that high IL-6, TNF-α, and IL-1β are predictive of poor leukemic outcomes, the synergic effect of these cytokines is likely to affect NK cell function. In addition to diminished cytotoxicity and tumor-killing, leukemic environments may push NK cells toward a regulatory phenotype noted in other inflammatory conditions (124). These skewed NK cells secrete IL-17, GM-CSF, TNFα, which could favor the growth of leukemia by acting directly on malignant cells. Defective IL−15 signaling is a potential driver of NK cell hypomaturation and poor survival in leukemia, as demonstrated in the context of solid tumors (125). This is supported by the fact that partial restoration of NK cell function is achieved in AML patients treated with soluble and IL15Rα-complexed IL-15 (126). Despite this success, adoptive NK cell immunotherapies require improvement to overcome the polarizing effects of disease-associated cytokines.

### CD4 and CD8 T Cells

Characteristically, the role of T cells is that of immune protection, surveillance, and clearance. The earliest bifurcation in T cell identity is the distinction between cytotoxic CD8^+^ T cells, responding to the presentation *via* MHC- class I and T-helper CD4^+^, activated by MHC-class II interactions. CD8^+^ T cells play critical roles in viral and cancer immunity and are defined by their capacity to kill and lyse malignant cells upon T-cell receptor (TCR) recognition. Helper CD4^+^ T-cell subsets are defined according to cytokine production, lineage-defining transcription factors, and more recently, transcriptional profile. Although recent advances in single-cell genomics and lineage tracing models have highlighted the incredible plasticity among the helper subsets, T helper (Th)1, Th2, Th17, Tregs, and follicular helper T cells (T_FH_) represent the five basic differentiation lineages (127).

Loss of T cell function is an emerging pathogenetic feature in myeloid malignancies. Gross alterations include dysfunctional CD8^+^ cytotoxic T-cells (CTLs), increased expression of immune checkpoint receptors and putative exhaustion markers such as Programmed cell death protein 1 (PD1), T cell immunoglobulin and mucin domain 3 (TIM3), and cytotoxic T-lymphocyte-associated protein 4 (CTLA4) in both CD4^+^ and CD8^+^ T-cells. Overexpression of leukemia-associated antigens such as proteinase-1 (PR1) and Wilm’s tumor-1 (WT1) have also been reported (128–130). In CML, dysfunctional T cell immunity is characterized by the absence of IFN-γ and TNF-α production (131, 132). One report also suggests that the cytokine response of T-cell subsets before treatment with chemotherapy or TIK is a predictive indicator of durable remission, highlighting the importance of endogenous T-cell function (132). Nevertheless, although defects in T cell-secreted cytokines, cytokine milieu, surface phenotypes, and transcriptional profile have been described, a more comprehensive characterization of the T cell landscape is needed.

Immune-altering effects of TKI-therapy on the T cell compartment have also been reported. Some groups report treatment with the TKI Imatinib inhibits T cell proliferation and activation (133). It has also been reported that treatment with different TKIs, such as Dasatinib, can trigger clonal T cell expansion, indicative of leukemic antigen recognition and anti-CML cytotoxicity (134). The PD1- PD-L1 pathway has also been implicated in CML pathology. In T cells, PD-1 is a co-inhibitory receptor, upregulated by activated T cells, and its ligation with PD-L1 is critical for immune tolerance and downregulation of immune responses. Dysregulated PD-1-PD-L1 signaling has been detected in T cells from CML patients along with increased expression of exhaustion-associated markers (TIM3, CTLA4) (128, 131). Ongoing research has focused on identifying leukemia-associated antigens expressed to develop immunotherapy. However, functional targets that elicit a robust repose are still lacking, and the effect of the inflammatory cytokine environment on T cell activation presents another obstacle.

### Regulatory T Cells

Tregs are critical for the preservation of immunological homeostasis. Principally they suppress the proliferation and cytokine production of effector T cells and myeloid populations (135). While the impact of Tregs has been readily explored in the context of autoimmunity and solid tumors, their contribution to hematological malignancies has not. Moreover, while it is known that Treg abnormalities accompany leukemic transformation, the mechanisms lack resolution. This is true of MDS, where an association has been established between Treg dysregulation and disease onset, but the pathogenic link is unknown (136). There is also a correlation between MDS and autoimmune diseases, further indicating a defect in Treg function. In line with this, elevated serum levels of IFN-γ, IL-17, IL-12, and RANTES, along with an increase in the Th17/Tregs ratio, have been reported in low-risk MDS, substantiating associations autoimmunity (137). Tregs also have blunted proliferation and reduced cell-cycle progression during the early stages of MDS, accompanied by a heightened expansion of IL-17 and GM-CSF secreting Th17 cells in the marrow of low-risk MDS patients (138).

The roles of Tregs span beyond immunosuppression and a growing body of evidence now supports the critical role for distinct “tissue Treg” subpopulations in tissue homeostasis (139). Within the bone marrow, Tregs have unique supportive functions related to HSC maintenance, and they are noticeably increased in frequency compared to other lymphoid tissues (~30-40% of CD4^+^ T cells). Studies have shown that bone marrow Tregs maintain stromal cells and the support hematopoiesis *via* enhanced IL-10 secretion. This cooperative relationship between Tregs and the stromal niche relies on a unique dependency of mesenchymal stromal cells on Treg secreted IL-10 which enhances hematopoietic support in a STAT3 dependent mechanism (140).

Data are conflicting regarding Treg alterations in MPN patients, as different studies report either increased or decreased numbers of Tregs in peripheral blood of patients. This is further complicated by the changes in Tregs that accompany treatment with TKIs, demethylating agents, and chemotherapeutic agents (141). On study found that patients with more BCR-ABL transcripts have increased Treg frequencies (142). Conversely, others have shown that Tregs are elevated in CML patients at the time of diagnosis but decrease dramatically in patients with chronic phase CML (143). This suggests that different phenotypes are pertinent at different phases of CML, highlighting the need to profile Tregs in different patient populations, at different disease stages, and following therapy. Broadly, the contribution of Tregs to the process of oncogenesis has been regarded as deleterious. It is well documented that the accumulation of immunosuppressive Tregs contributes to immune evasion and decreased immunosurveillance in a wide spectrum of cancers, effectively suppressing effective anti-tumor immunity (144). In myeloid malignancies, Tregs are similarly associated with a more aggressive disease or with an increased risk of relapse or drug resistance. However, most of these studies analyzed Tregs at later stages of the disease or after significant therapeutic intervention. Thus, there is limited information on how the Treg profiles change in proportion to disease progression. A basic question that needs to be addressed is whether Tregs are functional in patients with early disease. Nonetheless, a few reports have emerged detailing a beneficial prognosis for patients with higher Treg numbers (145, 146). Defining how Treg functions, including IL-10 secretion, are lost during leukemic transformation are outstanding questions.

**Figure 2:**
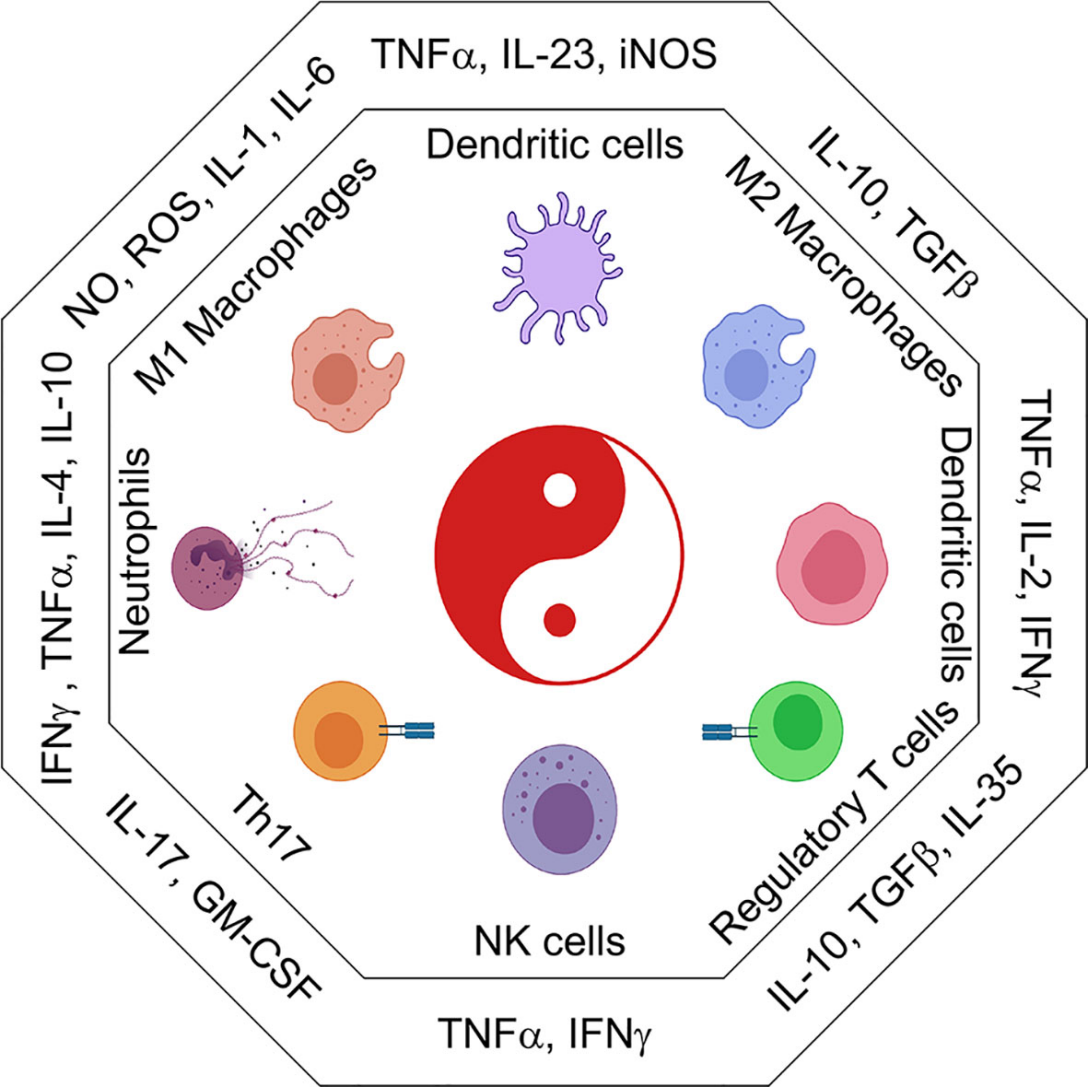
The immune cell universe in myeloid malignancies. Schematic representation of adaptive and innate immune subsets and their associated cytokines and secreted factors. Depending on the cytokine context immune cells take on distinct effector and regulatory profiles some are good, some are bad. These functions are characterized by the secretion of pro-inflammatory (IL1β, IL6, TNFα, CSFs) or suppressive (IL10, TGFβ, IL35) cytokines. Uncontrolled regulation of cytokines can result in the development of inflammatory leukemic disease.

*Refer to* **Figure 2** *for inflammatory cytokines and corresponding immune populations.*

### Immune Secreted Extracellular Vesicles

Extracellular vesicles (EVs) have emerged as potent immunomodulatory factors, both in the steady state and disease (147). Various subclassifications of EVs have been reviewed, but they are broadly defined as submicron membrane-bound lipid vesicles shed by most cells into the extracellular milieu. The specific function of EVs depends on the cell of origin and the associated cargo which can include proteins, nucleic acids (DNA, miRNA, mRNA), and lipids. The three main EV classifications are apoptotic bodies, microvesicles, and exosomes. Exosomes influence critical processes such as antigen presentation, immune activation and suppression, and metastatic transformation. They also have pivotal roles in the education of tissue niches and tumor microenvironments (148). Because the state of the immune cell is a critical determinant of EV cargo, it is likely that leukemic immune remodeling results in more inflammatory EV profiles. DCs pre-treated with LPS release EVs with increased levels of MHC Class-II, CD86 and ICAM1 molecules thus acting as more potent stimulators of T-cell function (149). Macrophages and DCs also release IL-1β, *via* EVs, and those harboring somatic mutations likely release more EV-loaded IL-1β than non-mutant counterparts. Various TNF superfamily members, including FasL, TRAIL and CD40L are also carried in exosomal membranes, and likely exacerbate targeting *via* NK cells and CTLs (150, 151). From the immune inhibition perspective tumor-derived exosomes can deliver functional PD-L1 thereby inhibiting immune responses and promoting immune escape (152). In myeloid malignancies, EVs likely play important roles at multiple stages of leukemogenesis, from suppressing the anti-leukemic immune responses to facilitating formation of inflammatory feedback loops. Understanding how specific clonal mutations shape the EVs landscape to regulate immunity, and how the role of EVs in disease trajectory should be carefully considered from a biomarkers and therapeutic standpoint.

## Clinical Targeting of Cytokines and Inflammatory Signals and Immunotherapeutic Approaches

In myeloid malignancies, chronic inflammation promotes disease progression, but temporal dynamics and cellular sources of these remain elusive. Nonetheless, therapies that target the cytokines themselves or downstream signaling pathways have had some efficacy in reducing disease-related pathologies. The adverse effects of cytokine signaling cascades in perpetuating cellular inflammation, leukemic transformation, and myeloproliferation have prompted cytokine therapy for clinical applications. Although cytokine-based immunotherapy is often a multi-edged sword, targeting IL-6 has shown efficacy. In CML, targeting of IL-6 with neutralizing antibodies had a protective effect against transformation demonstrating this cytokine’s centrality as an independent risk factor and driver of disease (45, 46). In AML, IL-6 blockade reversed bone marrow failure and restored erythroid differentiation (153). Genetic analyses further substantiated this in which loss of function polymorphisms in the IL-6 receptor reduces the risk of somatic transformation and progression (154).

IFN administration has achieved mixed clinical success. In MDS, early investigations documented that prolonged exposure to IFN reduced instances of infection, bleeding events, and progression to more advanced disease. Notably, clinical parameters reverted upon IFN-α withdrawal (155, 156). In *JAK2V617F*-mutant MPN and PV patients, long-term IFNα therapy has had success in promoting molecular remission and decreasing the JAK2 allelic burden (157, 158). However, in CML, prolonged treatment with IFN-α upon TKI withdrawal has been associated with expansion of immunoregulatory cells and lower frequencies of mature CD56dim NK cell (159). Similar observations have been made in JAK2-positive MPNs, where IFN-α treatment led to the expansion of regulatory CD56br NK subset and had no association with positive outcomes (160).

Hyperactivation and dysregulation of JAK/STAT pathways are universal, either by the presence of *JAK2V617F* mutations, oncogenic fusions that constitutively activate tyrosine kinase pathways, or the overexpression of cytokines (IL-6, IL-1β) that signal through these mechanisms. Thus, the beneficial effects of tyrosine kinase inhibition in ameliorating symptoms are manifold and represent the standard of care. Undoubtedly, these approaches also blunt and restrict the activation and function of most immune populations. Therefore, off-target effects into homeostatic immune functions such as wound healing, tissue niche maintenance, and metabolic processes should also be carefully weighed against the potential anti-leukemic effects.

Clearly, decreasing inflammatory factors and tempering the uncontrolled growth of leukemic cells is necessary. However, more investigations are needed into how ablating immune function may license more aggressive cancer development or equally damaging pathologies such as increased infection susceptibility. Leukemia-driven alterations in NK cells are of particular interest due to their anti-cancer cytolytic potential. Transferring ex vivo pre-activated NK cells is being employed in AML and recently in MDS, with a supportive cytokine treatment given to override the suppressive tumor environment, although the first results in AML trials appear disappointing (161). These adoptively transferred NK cells may acquire the traits of dysfunction akin to the endogenous patients’ NK cells due to the exposure to the leukemic microenvironment. Moreover, the leukemic inflammation may contribute to the hypo-responsiveness of adoptive NK cells to IL-15. In addition, dissecting the temporal effects of leukemic cytokines on NK cell maturation could be a potent way to boost endogenous NK cell responses and sensitize these cells to supportive cytokine therapies. In CML, boosting NK cell compartment, during or post-TKI, may help redirect the immune system toward eradicating residual leukemic cells and prevent the relapse (115, 118). Importantly, some of TKIs inhibit T cell function but do not seem to alter NK cell cytotoxicity and cytokine secretion, allowing for combinatory treatment (162).

The use of cellular therapies is still at its infancy in myeloid malignancies, but strategies to harness immune function are underway. In solid tumors, checkpoint blockade therapy that re-invigorates T cells has revolutionized cancer treatment. The immune checkpoint inhibitors have provided a path to reverse tumor-induced T-cell suppression and to restore anti-tumor activity. In AML, immune checkpoint molecules (TIM3, LAG-3, PD-1) are co-expressed in T cells, and there is significant expansion of the effector T cell pool, indicative of exhaustion. Reactivation of this pool through immune checkpoint blockade may be beneficial for anti-leukemic responses, although the main consideration would be to retrain excessive cytokine responses in an already inflammatory setting.

The specificity of the adaptive arm has limited its therapeutic efficacy in myeloid malignancies where mutational burden and immunogenicity is low, and few leukemia-specific antigens have been identified. Interestingly, the clinical targeting of the macrophage checkpoint CD47(an anti-phagocytic integrin associated protein) is becoming a promising therapeutic target. CD47 functions as a “do not eat me” signal and is upregulated by both normal and neoplastic cells to avoid phagocytic targeting. The overexpression of CD47 as a tumor evasion strategy was initially reported in AML and has since been described in MDS and other hematological and solid cancers (163). In homeostatic conditions, cells also upregulate pro-phagocytic “eat me” signals, such as calreticulin, in order to orchestrate programmed cell removal. The success of CD47 therapy relies on the absence of pro-phagocytic “eat me” signals in normal cells, which allows for selective targeting of malignant cells that typically co-express both signals. Although more work is needed to fine-tune cell type specific vulnerabilities to elimination by CD47 blockade, as is seen in red blood cells (RBCs), several approaches that target the CD47 pathway which block CD47 or its corresponding macrophage receptor SIRPα are underway (164). These represent exciting implementations of immunotherapy, particularly as part of combinatorial treatments (165).

For MDS and AML patients, epigenetic drugs such as hypomethylating agents (5-azacytidine (AZA) 5-aza-2′deoxycytidine, and others) remain the most effective intervention for improving prognosis and delaying progression. These agents function as DNA methyltransferase inhibitors and promote demethylation of DNA as well as de-condensation of the chromatin (166). These epigenetic therapies also have strong modulating effects on immune cell differentiation, gene expression, and function. In T cells, epigenetic modifications are a dominant mechanism for reinforcing effector fates into different Th-subsets. Treg lineage stability is likewise particularly sensitive to DNA methylation patterns and epigenetic regulation. The specific effects of hypomethylating agents on NK cells vary but mounting evidence points to alterations to promotor methylation of KIR genes and subsequent KIR expression. The impact of azacytidine on CD40 and CD86 expression on mature DCs has also been reported. In macrophages, polarization between M1 and M2 subsets, and cytokine secretion is similarly governed by epigenetic regulators, specifically DNA methyltransferases (167). Interestingly, the use of the DNMT inhibitor, decitabine, during differentiation increased phagocytosis, promoted M2 phenotypes, and reduced the proinflammatory cytokine secretion (168). Untangling the clinical benefits of epigenetic drugs on specific immune cell functions is a complex undertaking which will have to be done cautiously and in combination with systems-level approaches that integrate transcriptomic and proteomic measurements. Nonetheless, cataloging disease and stage-specific immune signatures will undoubtedly improve clinical outcomes and help predict immunotherapy responses in myeloid malignancies.

## Conclusions

The leukemic microenvironment is a stochastic niche in which cytokine interactions between immune cells and leukemic cells profoundly influence growth and metastatic spread. However, compared to solid tumors and autoimmune conditions, comprehensive profiling of the systemic immune cell dysfunction in myeloid diseases is lacking. Future work that dissects the effects of immune dysregulation on the stromal microenvironment has enthusing implications for developing therapeutic strategies that aim to rejuvenate niche function. Immune-mediated inflammation likely contributes to leukemic niche remodeling, “niche-facilitated” leukemic evolution, and disease permissive reprogramming of the stromal niche. As the crosstalk between mesenchymal stem and stromal cells with niche-occupying immune cells is critical for tissue health beyond hematopoiesis, strategies to preserve these homeostatic functions should be pursued.

The ubiquitous nature of leukemia in the body imposes distinctive immunological pressures and exciting challenges for evaluating the impact of oncogenic transformation on the host immune system. Unlike solid tumors, leukemias are widely disseminated throughout the body. This creates a scenario where immune cells, both in circulation and across tissues, are near neoplastic cells and face constant exposure to inflammatory cue., and it is likely that multiple tissue niches can support metastasis, as evidenced by extramedullary hematopoiesis. An outstanding question is whether we can take advantage of signaling differences in bystander and mutation-carrying immune cells to alter disease progression. At the more fundamental level, how are homeostatic immune functions affected by competition with mutant immune cells, and what are the implications of sharing a microenvironment for immune regulation more broadly?

Another area for a further investigation relates to the specific leukemia signals that affect T cell differentiation. The current body of work suggests that the leukemic environment co-opts the T cell compartment to favor its survival and propagation. In addition to Tregs alterations, it is likely that naïve T cells may have defects or biases to other T helper fates. Answers to these inquiries will require more comprehensive profiling of the key cytokines and chemokines in specific leukemic microenvironments and rigorous definitions of what constitutes short-term and long-term adaptation relative to inflammatory responses.

Immune alterations in myeloid malignancies are unified by a theme of excess inflammation, but more work is needed to clarify how immune cells shape leukemic evolution and on how the dose and duration of exposure to specific cytokines shape individual cell types. Dissecting this feedback will also aid the development of prophylactic therapies to prevent the inflammation-induced acceleration of leukemia to intervene in the earliest and most crucial stages of cancer development.

## Author Contributions

VC: proposed, drafted, and wrote this review. VK: drafted and wrote this review. RW: proposed, drafted, and wrote this review. All authors contributed to the article and approved the submitted version. Figure Created with assistance from BioRender.com.

## Funding

This article was supported by an NHLBI grant, 1R01HL150078.

## Conflict of Interest

The authors declare that the research was conducted in the absence of any commercial or financial relationships that could be construed as a potential conflict of interest.

## Publisher’s Note

All claims expressed in this article are solely those of the authors and do not necessarily represent those of their affiliated organizations, or those of the publisher, the editors and the reviewers. Any product that may be evaluated in this article, or claim that may be made by its manufacturer, is not guaranteed or endorsed by the publisher.
